# 182. Progress towards an EBV vaccine: Results of a Phase I, First-In-Human EBV gp350 Ferritin Nanoparticle Vaccine Candidate adjuvanted with Matrix-M®

**DOI:** 10.1093/ofid/ofaf695.061

**Published:** 2026-01-11

**Authors:** Jessica Durkee-Shock, Hanh Nguyen, Alexander C Vostal, Sally Hunsberger, Megan C Grieco, Krista Gangler, Karenna Barton, Maria Ploussiou, Kelly Liepshutz, Kayla Morgan, Anna Hostal, Meghna Bagchi, Leonid Serebryannyy, Mike Castro, Michael Sibilo, Masaru Kanekiyo, Lesia Dropulic, Kennichi Dowdell, Wei Bu, Jeffrey I Cohen

**Affiliations:** Laboratory of Infectious Diseases, National Institute of Allergy and Infectious Diseases, National Institutes of Health, Bethesda, Maryland; Laboratory of Infectious Diseases, National Institute of Allergy and Infectious Diseases, National Institutes of Health, Bethesda, Maryland; Laboratory of Infectious Diseases, National Institute of Allergy and Infectious Diseases, National Institutes of Health, Bethesda, Maryland; National Institute of Allergy and Infectious Diseases, Bethesda, Maryland; National Institute of Allergy and Infectious Diseases, Bethesda, Maryland; Laboratory of Infectious Diseases, National Institute of Allergy and Infectious Diseases, National Institutes of Health, Bethesda, Maryland; Laboratory of Infectious Diseases, National Institute of Allergy and Infectious Diseases, National Institutes of Health, Bethesda, Maryland; Laboratory of Infectious Diseases, National Institute of Allergy and Infectious Diseases, National Institutes of Health, Bethesda, Maryland; Frederick National Laboratory for Cancer Research, Leidos Biomedical Research Inc., Bethesda, Maryland; Laboratory of Infectious Diseases, National Institute of Allergy and Infectious Diseases, National Institutes of Health, Bethesda, Maryland; Laboratory of Infectious Diseases, National Institute of Allergy and Infectious Diseases, National Institutes of Health, Bethesda, Maryland; Laboratory of Infectious Diseases, National Institute of Allergy and Infectious Diseases, National Institutes of Health, Bethesda, Maryland; Vaccine Research Center, National Institute of Allergy and Infectious Diseases, National Institutes of Health, Bethesda, Maryland; Vaccine Research Center, National Institute of Allergy and Infectious Diseases, National Institutes of Health, Bethesda, Maryland; Vaccine Research Center, National Institute of Allergy and Infectious Diseases, National Institutes of Health, Bethesda, Maryland; Vaccine Research Center, National Institute of Allergy and Infectious Diseases, National Institutes of Health, Bethesda, Maryland; Laboratory of Infectious Diseases, National Institute of Allergy and Infectious Diseases, National Institutes of Health, Bethesda, Maryland; Laboratory of Infectious Diseases, National Institute of Allergy and Infectious Diseases, National Institutes of Health, Bethesda, Maryland; Laboratory of Infectious Diseases, National Institute of Allergy and Infectious Diseases, National Institutes of Health, Bethesda, Maryland; Laboratory of Infectious Diseases, National Institute of Allergy and Infectious Diseases, National Institutes of Health, Bethesda, Maryland

## Abstract

**Background:**

Epstein Barr virus (EBV) is a ubiquitous ץ-herpesvirus associated with mononucleosis, cancers, and autoimmune disease including multiple sclerosis. Currently, there is no FDA-approved vaccine for EBV. We previously designed a self-assembling EBV gp350 ferritin nanoparticle (FNP) vaccine which elicited potent EBV neutralizing titers in animal models. Here we present the results of the phase I, first-in-human EBV gp350 FNP vaccine clinical trial.

**Methods:**

Fifty μg of EBV gp350FNP vaccine adjuvanted with 50μg of Matrix-M®was administered in an open-label trial to 40 healthy young adults age 18-29 years. Twenty EBV seropositive (SP) and 20 EBV seronegative (SN) individuals received 3 doses of vaccine at days 0, 30, and 180. The composite primary endpoint consisted of 1) safety as assessed by unsolicited adverse events (AEs) and severe AEs through day 210, as well as local and systemic reactogenicity and 2) immunogenicity as assessed by change in log_10_ EBV neutralizing antibody titer from baseline to day 210.

**Results:**

Of 20 SN and 20 SP individuals enrolled, 16 SP and 19 SN individuals completed the primary endpoint at day 210. Participants experienced mild to moderate reactogenicity; the most common AEs were injection site pain and tenderness, fatigue, chills, and headaches. All expected AEs were self-limited and required minimal medications. There were no SAEs related to the study agent. The EBV gp350 FNP elicited a 16-fold and 67-fold increase in EBV neutralizing titers from baseline in SP and SN individuals, respectively (p< 0.05). At day 210, SN persons demonstrated 3.2-fold higher antibody titer than the baseline titers of convalescent SP persons. Total EBV gp350-binding titers measured by immunoprecipitation assay at day 0 and 210 were increased by 40-fold and 4-fold in SN and SP respectively (p< 0.05). The neutralizing titers in SN persons were durable from day 210 to day 360. No SN individual acquired EBV infection after completion of 3 doses of study agent through the last required study visit (day 540).

**Conclusion:**

The EBV gp350 FNP vaccine adjuvanted with Matrix M1 was safe, tolerable, and immunogenic in a phase I, first-in-human, open label clinical trial.

**Disclosures:**

All Authors: No reported disclosures

